# Three-dimensional locations of ruptured retinal arterial macroaneurysms and their associations with the visual prognosis

**DOI:** 10.1038/s41598-021-04500-4

**Published:** 2022-01-11

**Authors:** Saori Sakaguchi, Yuki Muraoka, Shin Kadomoto, Sotaro Ooto, Tomoaki Murakami, Naomi Nishigori, Masaharu Ishikura, Masahiro Miyake, Manabu Miyata, Akihito Uji, Akitaka Tsujikawa

**Affiliations:** Department of Ophthalmology and Visual Sciences, Kyoto University Graduate School of Medicine, Sakyo-ku, Kyoto, 606-8507 Japan

**Keywords:** Anatomy, Diseases, Pathogenesis

## Abstract

The aim of this retrospective, observational study was to examine the intraretinal locations of ruptured retinal arterial macroaneurysms (RMAs) and investigate the associations with the visual prognosis. Fifty patients (50 eyes) with untreated RMA rupture who visited the Department of Ophthalmology at Kyoto University Hospital (April 2014–July 2019) were included. The intraretinal position of the ruptured RMAs relative to the affected retinal artery was examined using optical coherence tomography (OCT) and color fundus photography (CFP). The relative RMA positions were anterior to (anterior type, 44%), at the same level as (lateral type, 20%), or posterior to (posterior type, 34%) the affected artery. At the initial visit, the posterior type showed greater subretinal hemorrhage thickness than did the lateral and anterior types (*P* = 0.016 and 0.006, respectively), and poorer visual acuity (VA) than did the anterior type (*P* = 0.005). At the final visit, the length of the foveal ellipsoid zone band defect was longer (*P* = 0.005) and VA was poorer (*P* < 0.001) for the posterior type than for the anterior type. The intraretinal positions of ruptured RMAs vary, affect the thickness of foveal subretinal hemorrhage and predict future damage to the foveal photoreceptors. The visual prognosis may be poor for posteriorly ruptured RMAs.

## Introduction

Retinal arterial macroaneurysm (RMA) is an acquired, focal dilation of a retinal artery, typically occurring within the first three bifurcations of the central retinal artery. It is relatively common in elderly women^1–4^. The pathophysiology of this disease is not completely understood; however, it is speculated that the decrease in the elasticity of the vessel wall due to arteriosclerosis may make it more susceptible to increased hydrostatic pressure. The diagnosis, in most cases, is made when a patient experiences loss of vision due to acute hemorrhagic changes or the subsequent exudative changes involving the macula.

Rupture of RMA can cause hemorrhages in the vitreous cavity, various layers of the retina, and subretina, with a concomitant sudden decrease in the visual acuity (VA). Treatment includes direct photocoagulation of RMA^5,6^, vitrectomy using internal limiting membrane (ILM) peeling or tissue plasminogen activator (t-PA)^7,8^, or intravitreal injections of an anti-vascular endothelial growth factor (VEGF)^9,10^. However, patients with irreversible poor vision despite these aggressive treatments are occasionally encountered.

In a previous study of patients with ruptured RMA who underwent vitrectomy with t-PA, the postoperative outcome was worse for patients with preoperative intraretinal hemorrhage than for patients without this hemorrhage^11^. However, the association of the visual prognosis with other types of hemorrhages has not been well studied. Moreover, there is little information regarding the relationship of the angiographic features and intraretinal locations of ruptured RMAs with the visual prognosis.

Therefore, the purpose of this study was to evaluate the positions of ruptured RMAs relative to the affected retinal artery using optical coherence tomography (OCT) and ophthalmoscopic findings, and to investigate the association of the intraretinal location with the pattern of retinal hemorrhage and visual prognosis.

## Methods

### Patients

This retrospective study was approved by the Institutional Review Board of Kyoto University Graduate School of Medicine (Kyoto, Japan) and adhered to the tenets of the Declaration of Helsinki. Written informed consent was obtained from each subject at the initial visit before study initiation.

The inclusion criterion was untreated RMA rupture, whereas exclusion criteria encompassed RMAs ruptured at a location more nasal than the optic disc, other ocular diseases (diabetic retinopathy, retinal vein occlusion, or uveitis), and patients with dense cataracts or vitreous hemorrhage that could degrade the image quality of OCT and interfere with the analysis. Eventually, 50 patients (50 eyes) with RMA rupture who visited the Department of Ophthalmology at Kyoto University Hospital between April 2014 and July 2019 met our eligibility criteria.

At the initial visit, in addition to a comprehensive ophthalmic examination including measurement of the best-corrected VA using a Landolt chart, 45° digital color fundus photography (CFP; TRC-50LX, Topcon, Tokyo, Japan; 3216 × 2136 pixels), fluorescein angiography, and indocyanine green angiography were performed after pupil dilation for each patient. Using OCT examinations (Spectralis HRA + OCT, Heidelberg Engineering, Heidelberg, Germany), we acquired horizontal and vertical scans through the center of the fovea, a 30° × 25° macular volume scan, and scans of RMA and the affected retinal artery. The VA measurement was performed at a distance of 5 m using a standard VA testing device (LED type) that complies with ISO 8596 and 8597. The brightness of the VA measurement room was 600 Lux, the illuminance of the optotype surface was 700 Lux, and the optotype contrast was approximately 98% (background luminance was approximately 240 cd/m^2^ and optotype luminance was approximately 3 cd/m^2^)^12^. In cases where the 0.1 index could not be answered correctly, VA was calculated based on the visual angle at the farthest distance where most correct responses to the 0.1 single optotype were obtained. RMA rupture was diagnosed by retinal specialists (YM, SO, TM) on the basis of the results of the comprehensive ophthalmic examination.

At each follow-up visit, the best-corrected VA was measured, and the macular area was examined using OCT and CFP for each patient.

### Anatomical positions of the ruptured aneurysm relative to the affected retinal artery

The anatomical position of RMA relative to the affected retinal artery was determined from both OCT scans and CFP images of RMA and the affected retinal artery (see supplementary Fig. 1 online). We used OCT scans of RMA to identify the position of RMA in relation to the affected retinal artery. We additionally used CFP images obtained at 3 to 12 months after rupture, when the vessel walls of RMAs became white and organized. Accordingly, RMAs located above or anterior to the affected artery, those located below or posterior to the affected artery, and those located at the same level as the affected artery, which could not be classified as the anterior or posterior type, were classified as anterior, posterior, and lateral RMAs, respectively.

### OCT evaluations of the hemorrhage pattern, foveal thickness, thickness of foveal subretinal hemorrhage, and length of the ellipsoid zone band defect

We also judged hemorrhagic patterns as vitreous, preretinal, sub-ILM, intraretinal, and subretinal hemorrhages using ophthalmoscopic findings, including CFP findings, and OCT scans passing through the fovea and RMAs [see Supplementary Fig. 2 online, which shows the different hemorrhage patterns in an eye with a ruptured retinal arterial macroaneurysm using optical coherence tomography; a: Vitreous hemorrhage (anterior to the posterior vitreous membrane), b: Preretinal hemorrhage (anterior to the inner limiting membrane and posterior to the posterior vitreous membrane), c: Sub-inner limiting membrane hemorrhage (confined below the inner limiting membrane), d: Intraretinal hemorrhage (inside the sensory retina), e: Subretinal hemorrhage (beneath the sensory retina)].

To measure the foveal thickness, a macular volume scan was acquired at each visit. For OCT, a whole-retinal thickness map centered on the foveal center was created using the Early Treatment Diabetic Retinopathy Study grid. The foveal thickness was defined as the average value calculated from the retinal thickness of the central grid. OCT measurements of the entire retinal thicknesses of each grid were performed using the manufacturer’s built-in software (Spectralis Acquisition and Viewing Modules, version 6.0, Heidelberg Engineering). We used automatic segmentation in Spectralis to evaluate the retinal thickness; however, if there was a segmentation error, we manually corrected the segmentation in that area to ensure that the retinal thickness could be evaluated correctly. Furthermore, at the first visit, the thickness of foveal subretinal hemorrhage was measured on vertical and horizontal OCT scans through the fovea, and the averaged value was used for analysis.

To assess the integrity of the foveal photoreceptor layer at the final visit, we quantified the disruption of the ellipsoid zone (EZ) band within the central 2-mm area on OCT images sectioned vertically and horizontally through the center of the fovea, and calculated the average value for each patient. The signal intensity of the EZ band was measured, and the EZ band was quantified using the plot profile function in ImageJ software (National Institutes of Health, Bethesda, MD, USA). According to previous reports^13^, the length of the EZ band defect was defined as the line on the grayscale image along which the EZ reflectivity diminished by two standard deviations relative to the reflectivity in the unaffected retina.

### Classification of the bifurcation order from the central retinal artery

Using the CFP images, we examined the order of the bifurcation (first to the third order) from the central retinal artery at the RMA site.

### Measurement of the distance between RMA and the fovea

Using infrared imaging in the Spectralis HRA + OCT device, the distance between RMA and the fovea was measured at 3–12 months after rupture, when the associated hemorrhages were substantially absorbed.

### Statistical analysis

Statistical analysis was performed using PASW Statistics version 18.0 (SPSS, Chicago, IL). Values are presented as the mean ± standard deviation. For statistical analysis, VA measured with a Landolt chart was converted to the logarithm of the minimum angle of resolution (logMAR) unit.

Comparisons among the three different groups were adjusted for multiple testing by the Bonferroni method. One case with an unknown aneurysm location was excluded from the analysis. Significant differences in the sampling distributions were determined using chi-square tests.

To determine the coefficient of correlation between the final VA and other clinical findings, we used Pearson’s product-moment correlation. Stepwise forward multivariate linear regression analysis was performed to evaluate the associations of the angiographic features (intraretinal location of RMA and distance between RMA and the foveal center) and gender with the final VA and foveal subretinal hemorrhage thickness. A *P*-value of < 0.05 was considered statistically significant.

## Results

This study included 50 eyes with RMA rupture (11 men and 39 women; mean age, 79.1 ± 7.8 years). Table 1 shows the clinical characteristics of the included patients. At the initial visit, the mean duration of symptoms from onset was 12.2 ± 17.4 days (range: 1–35 days), mean logMAR VA was 0.77 ± 0.58 (Snellen equivalent: 20/2000–20/16), and mean foveal thickness was 540 ± 276 μm. Twenty-two eyes had phakia; however, there were no patients with dense cataracts or vitreous hemorrhage, which could degrade the image quality of OCT and interfere with the analysis.

**Table 1:** Characteristics of patients with ruptured retinal macroaneurysms.

Number of patients (men/women)	11/39
Mean age, years	79.1 ± 7.8
Systemic hypertension, n (%)	35 (70)
Diabetes mellitus, n (%)	2 (4)
Smoking, n (%)	10 (20)
**At initial visit**	
Duration from the onset (days)	12.2 ± 17.4 Range 1–35
LogMAR visual acuity	0.77 ± 0.58
Snellen equivalent (range)	20/2000–20/16
Foveal thickness (μm)	540 ± 276
**Treatment, n (%)**	
Direct photocoagulation	8 (16)
Direct photocoagulation and anti-vascular endothelial growth factor therapy	7 (14)
Anti-vascular endothelial growth factor therapy	9 (18)
Pars plana vitrectomy	18 (36)
Sulfur hexafluoride gas injection	3 (6)
Only observation	5 (10)
**At the final examination**	
Duration from the onset (months)	22.4 ± 22.9
LogMAR visual acuity	0.34 ± 0.36
Snellen equivalent, range	20/200–20/16
Foveal thickness (μm)	292 ± 111
Length of the ellipsoid zone band defect (μm)	489 ± 569

LogMAR = logarithm of the minimal angle of resolution. Data are expressed as mean ± standard deviation unless otherwise indicated.

The follow-up period was 22.4 ± 22.9 months. As treatment for the ruptured RMA, eight eyes received direct laser photocoagulation, seven received both direct photocoagulation and intravitreal injections of anti-VEGF agents, nine received intravitreal injections of anti-VEGF agents, three received intravitreal injection of sulfur hexafluoride gas, and 18 received pars plana vitrectomy (with ILM peeling and t-PA injection as necessary) during the observational period. The remaining five eyes did not receive any treatment during the follow-up period.

At the final examination, the mean logMAR VA was 0.34 ± 0.36 (Snellen equivalent: 20/200–20/16), mean foveal thickness was 292 ± 111 μm, and length of the EZ band defect was 489 ± 569 μm (Table 1).

### Angiographic features of the ruptured retinal arterial macroaneurysms

The order of the bifurcation from the central retinal artery at the RMA site was as follows: first order, 4 (8%) eyes; second order, 28 (56%) eyes; and third order, 18 (36%) eyes. The distance from RMA to the fovea was 2774 ± 984 μm (range: 774–4849 μm, Table 2).

**Table 2:** Angiographic features of ruptured retinal macroaneurysms.

**Bifurcation order from the central retinal artery: n, (%)**	
First order	4 (8)
Second order	28 (56)
Third order	18 (36)
**Depth of the RMA lesion relative to the affected retinal artery, n (%)**	
Anterior, n (%)	22 (44)
Lateral, n (%)	10 (20)
Posterior, n (%)	17 (34)
Unknown, n (%)	1 (2)
Distance between the RMA and the fovea (μm)	2774 ± 984 Range 774–4849

RMA = retinal arterial macroaneurysm.

According to the three-dimensional evaluations using OCT and CFP images, 22 (44%), 10 (20%), and 17 (34%) RMAs were classified as the anterior, lateral, and posterior types, respectively (Table 2). For the remaining RMA (2%), we were unable to determine the three-dimensional intraretinal location relative to the affected artery; this case was excluded from the statistical analysis.

### Hemorrhagic patterns and the association with the aneurysm location

At the initial visit, vitreous hemorrhage was observed in 10 (20%) eyes, preretinal hemorrhage in three (6%) eyes, sub-ILM hemorrhage in 32 (64%) eyes, intraretinal hemorrhage in 50 (100%) eyes, and subretinal hemorrhage in 46 (92%) eyes.

**Figure 1:**
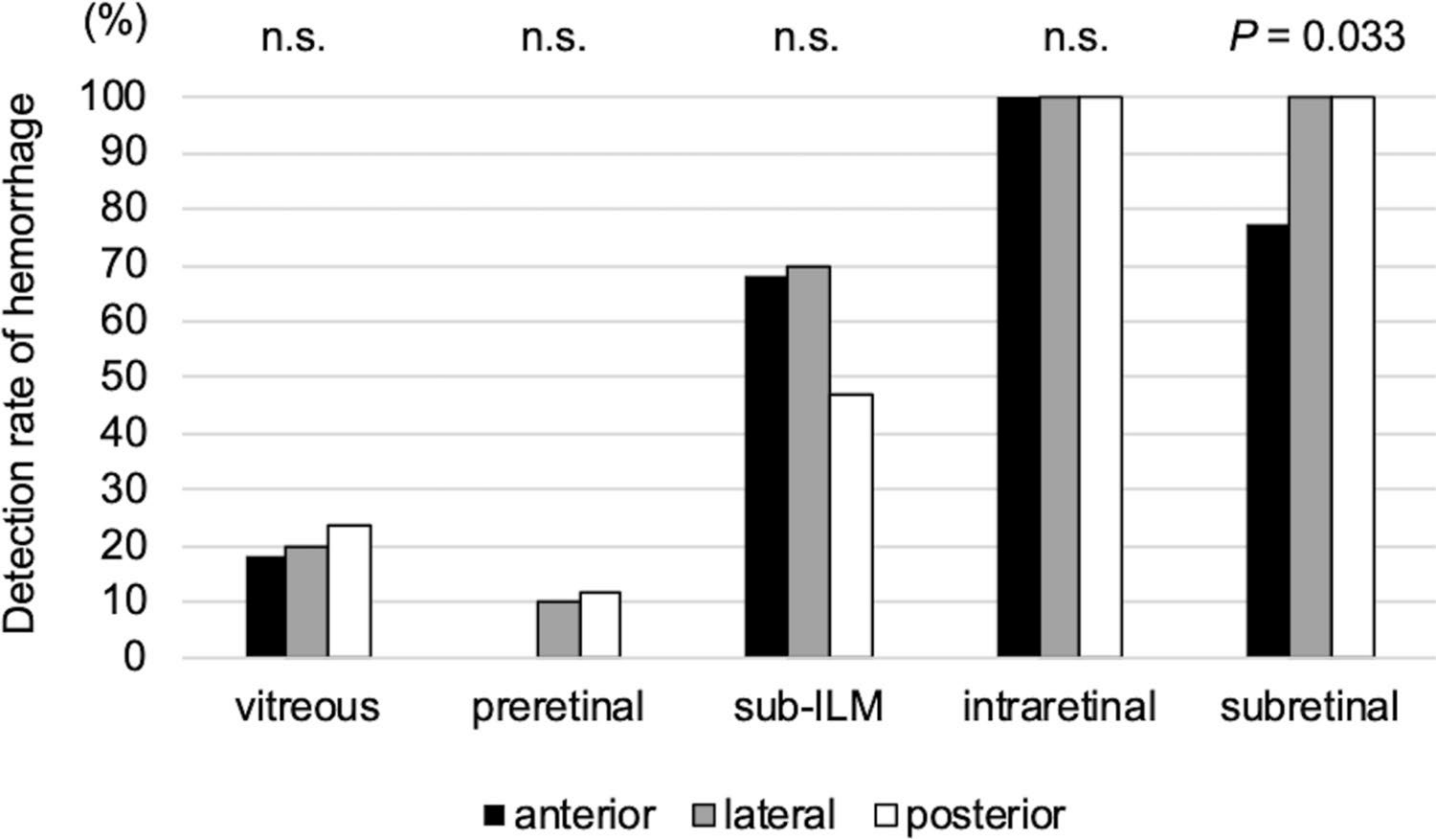
Association between the hemorrhagic pattern and the position of ruptured retinal arterial macroaneurysms relative to the affected retinal artery. The detection rate for sub-inner limiting membrane hemorrhage was not significantly associated with the depth of the ruptured retinal arterial macroaneurysm; however, the detection rate for subretinal hemorrhage was significantly lower for eyes with anteriorly ruptured RMAs (*P* = 0.033).

We examined the associations between each hemorrhage pattern and the intraretinal location of RMAs; the detection rate for sub-ILM hemorrhage was not significantly associated with the depth of RMA, whereas that of subretinal hemorrhage was significantly lower in eyes with anteriorly ruptured RMAs (*P* = 0.033, chi-square test, Fig. 1).

### Associations between the final visual acuity and other clinical findings

Table 3 shows the clinical parameters associated with the final visual outcome in the univariate analysis. The final logMAR VA showed a significant positive association with the initial logMAR VA (*P* = 0.006) and the distance between RMA and the fovea (*P* = 0.005). Among the initial anatomical parameters, the thickness of foveal subretinal hemorrhage showed the strongest association with a poor final visual outcome (*P* = 0.010).

**Table 3:** Association between the final visual acuity and other clinical findings examined by Pearson’s product-moment correlation.

	R	*P* value
Age	0.023	0.877
Time from the onset to the initial visit	0.041	0.803
**Baseline**		
LogMAR visual acuity	0.394	0.006
Foveal thickness	0.298	0.042
Thickness of foveal subretinal hemorrhage	0.373	0.010
Distance between RMA and the fovea	− 0.407	0.005
**Final visit**		
Foveal thickness	− 0.278	0.061
Defect length of the ellipsoid zone band	0.733	< 0.001

LogMAR = logarithm of the minimal angle of resolution. Data are expressed as mean ± standard deviation unless otherwise indicated.

Therefore, to determine the effects of angiographic features of RMAs on the visual outcome and thickness of foveal subretinal hemorrhage, we performed multivariate analysis with the intraretinal RMA location, the distance between RMA and the fovea, and gender as explanatory variables. In both analyses, the intraretinal locations of RMAs were more strongly associated with the final VA (β = 0.535, *P* < 0.001) and thickness of foveal subretinal hemorrhage (β = 0.462, *P* = 0.001).

### Associations between the intraretinal locations of the aneurysms and other clinical findings

**Figure 2:**
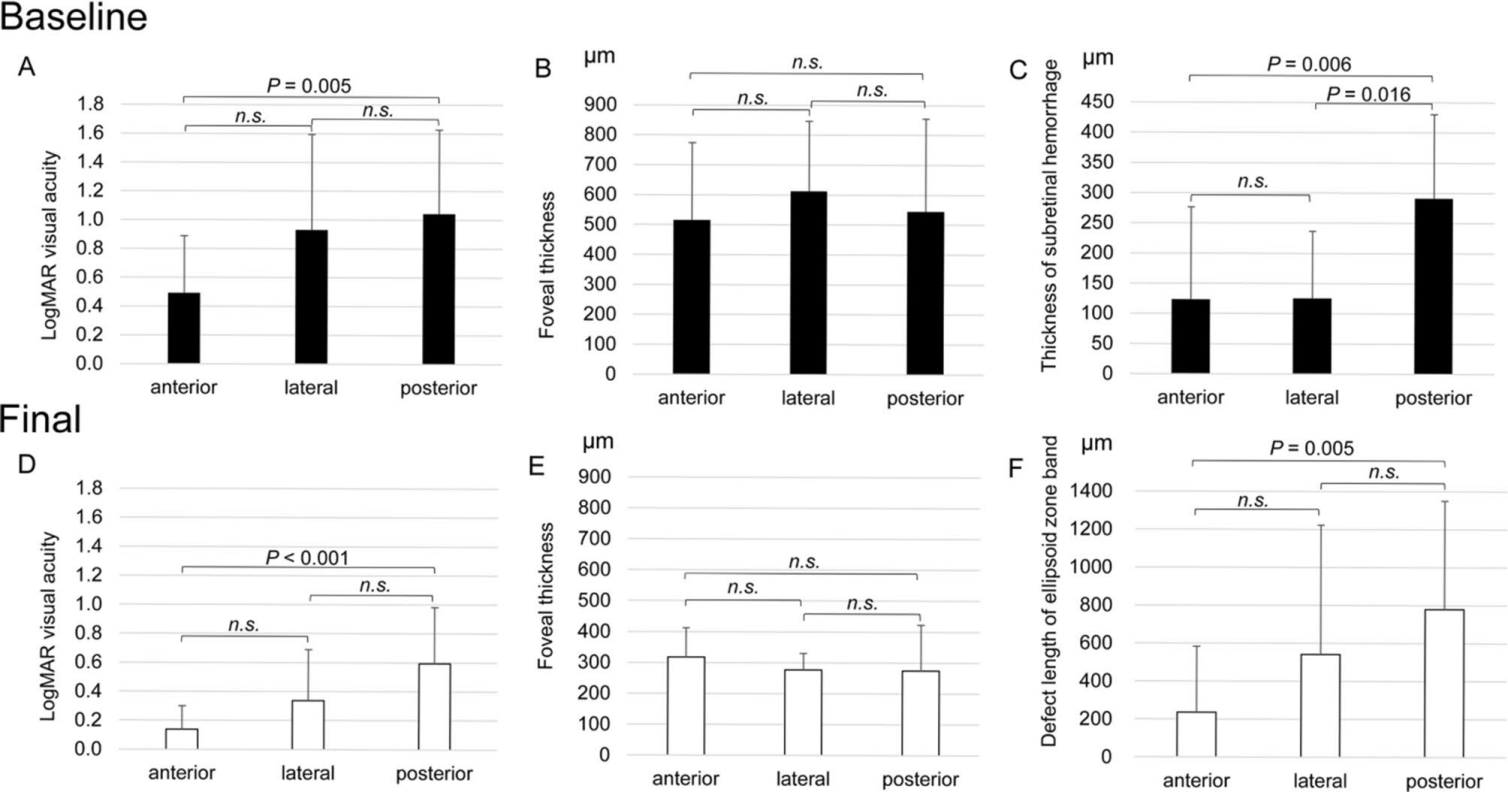
Baseline and final foveal function and morphology according to the position of ruptured retinal arterial macroaneurysms relative to the affected artery. (**A**,**B**,**C**) Baseline findings. (**D**,**E**,**F**) Final findings. (**A**,**D**) Logarithm of the minimum angle of resolution (LogMAR) visual acuity (VA). (**B**,**E**) Retinal thickness at the fovea. (**C**) Thickness of subretinal hemorrhage at baseline. (**F**) Length of the ellipsoid zone band defect. VA and the subretinal hemorrhage thickness at baseline are poorer and greater, respectively, for patients with ruptured retinal arterial macroaneurysms (RMAs) located posterior to the affected artery (posterior type) than for patients with ruptured RMAs located anterior to the affected artery (anterior type). At the final visit, the length of the foveal ellipsoid zone band defect is greater and the final VA is poorer for patients with the posterior type.

**Figure 3:**
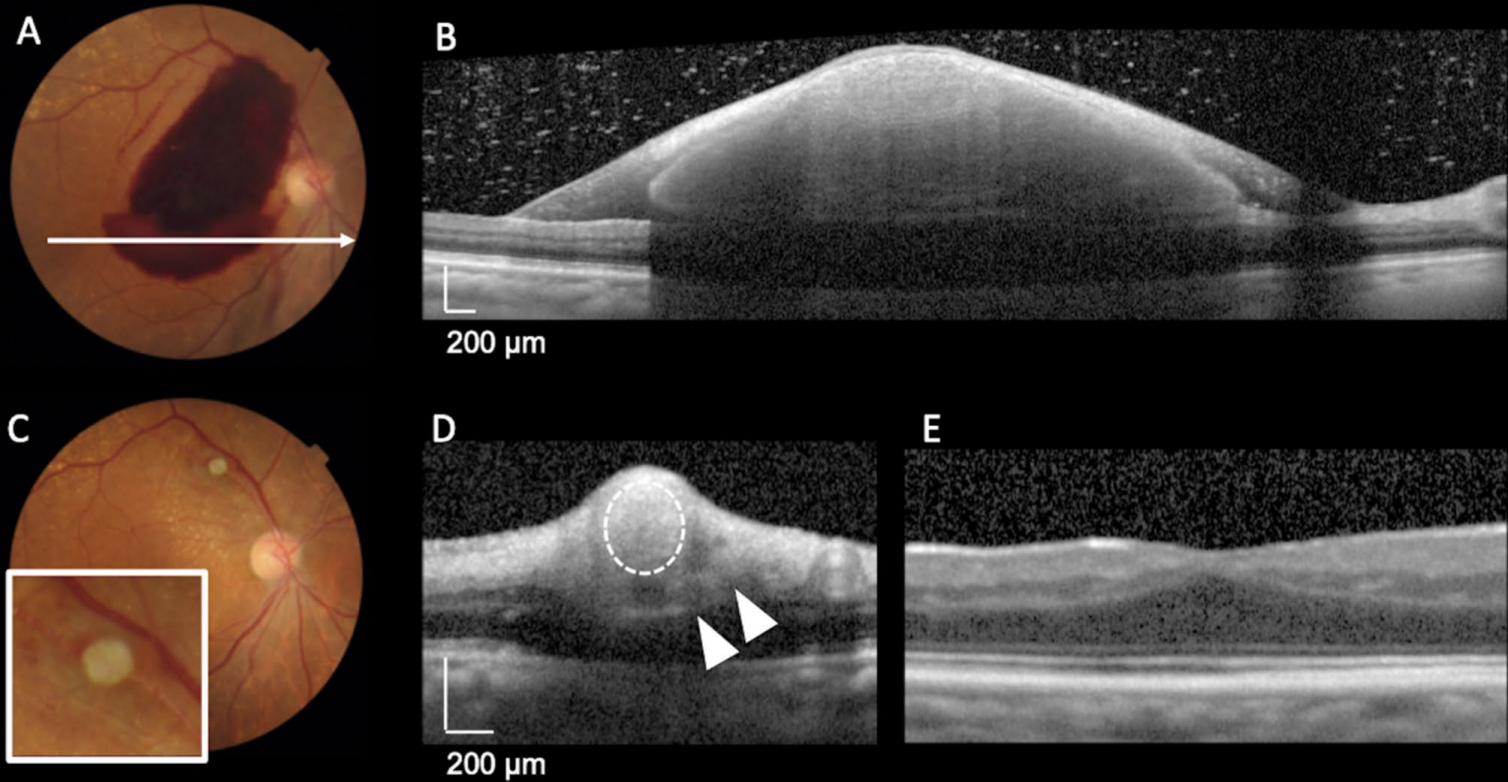
Representative case involving an 86-year-old woman with a ruptured retinal arterial macroaneurysm anterior to the affected artery (anterior type) in the right eye. Optical coherence tomography (OCT) performed in the acute phase shows a small amount of vitreous hemorrhage and a large amount of preretinal hemorrhage and sub-inner limiting membrane (ILM) hemorrhage. Subretinal hemorrhage is unremarkable (**B**). Eight days after onset, pars plana vitrectomy (PPV) with ILM peeling and direct photocoagulation of RMA were performed. On resolution of the hemorrhages, OCT shows that RMA is located anterior to the retinal artery (**D**, arrows), and color fundus photography (**C**) shows that the organized white RMA is interrupting the course of the affected artery. Thus, it can be classified as the anterior type. After 24 months, there is no damage to the foveal photoreceptor layer (**E**); the visual acuity was 20/16.

**Figure 4:**
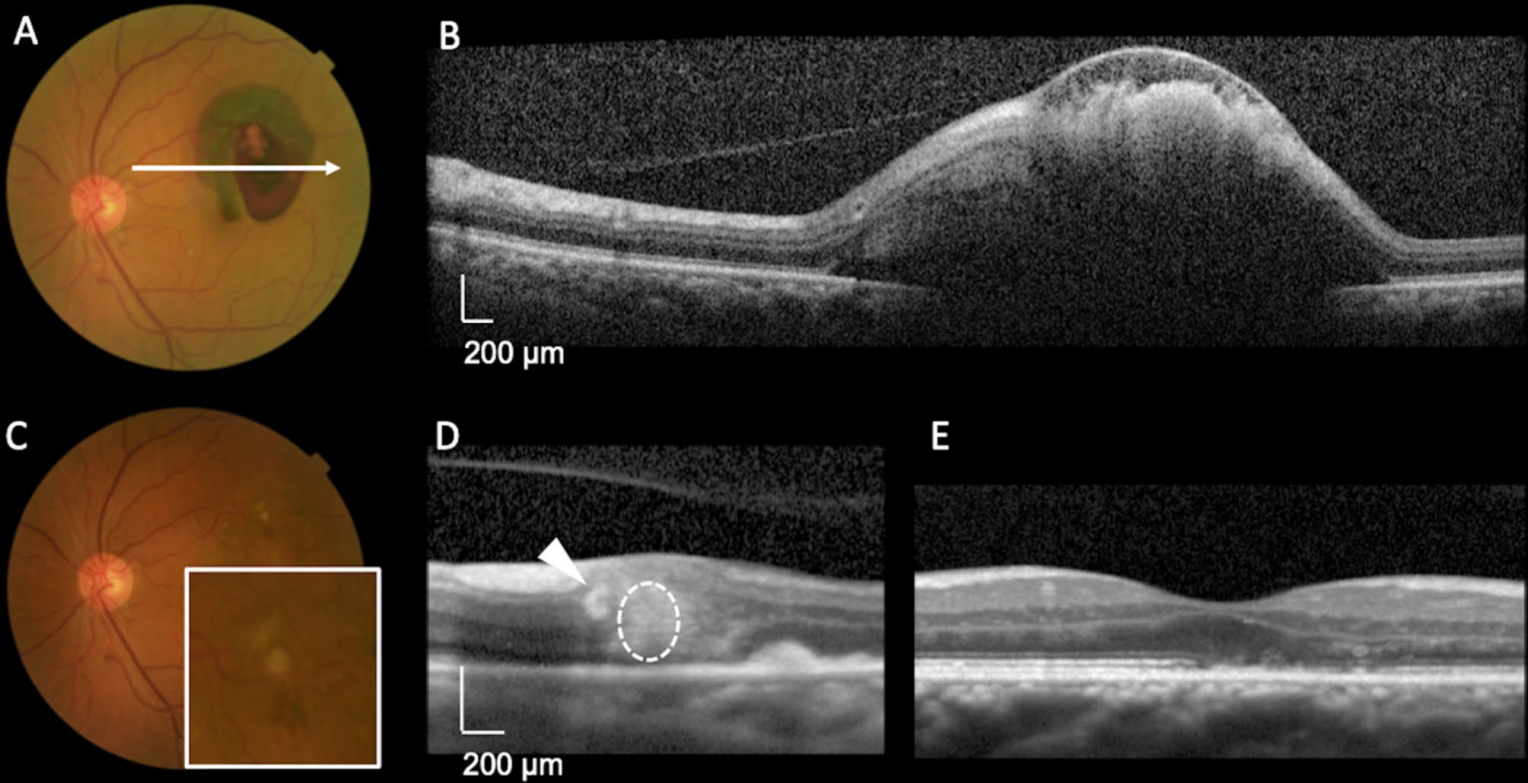
Representative case involving a 71-year-old man with a ruptured retinal arterial macroaneurysm at the same level as the affected artery (lateral type) in the left eye. At the initial visit, the visual acuity was 20/16. Optical coherence tomography (OCT) obtained in the acute phase shows sub-inner limiting membrane (ILM) hemorrhage and subretinal hemorrhage around the retinal arterial macroaneurysm (RMA) (**B**). The thickness of the foveal subretinal hemorrhage was 149 μm. At 5 days after onset, we treated RMA with direct photocoagulation and intravitreal injection of ranibizumab. A color fundus photograph (**C**) shows RMA protruding toward the center of the fovea without interfering with the course of the affected artery. OCT (**D**) shows RMA (dotted circle) located at approximately the same level as the artery (arrow). Thus, it was classified as the lateral type. At 24 months after onset, the length of the ellipsoid zone band defect at the fovea was 817 μm (**E**), and the visual acuity was 20/25.

**Figure 5:**
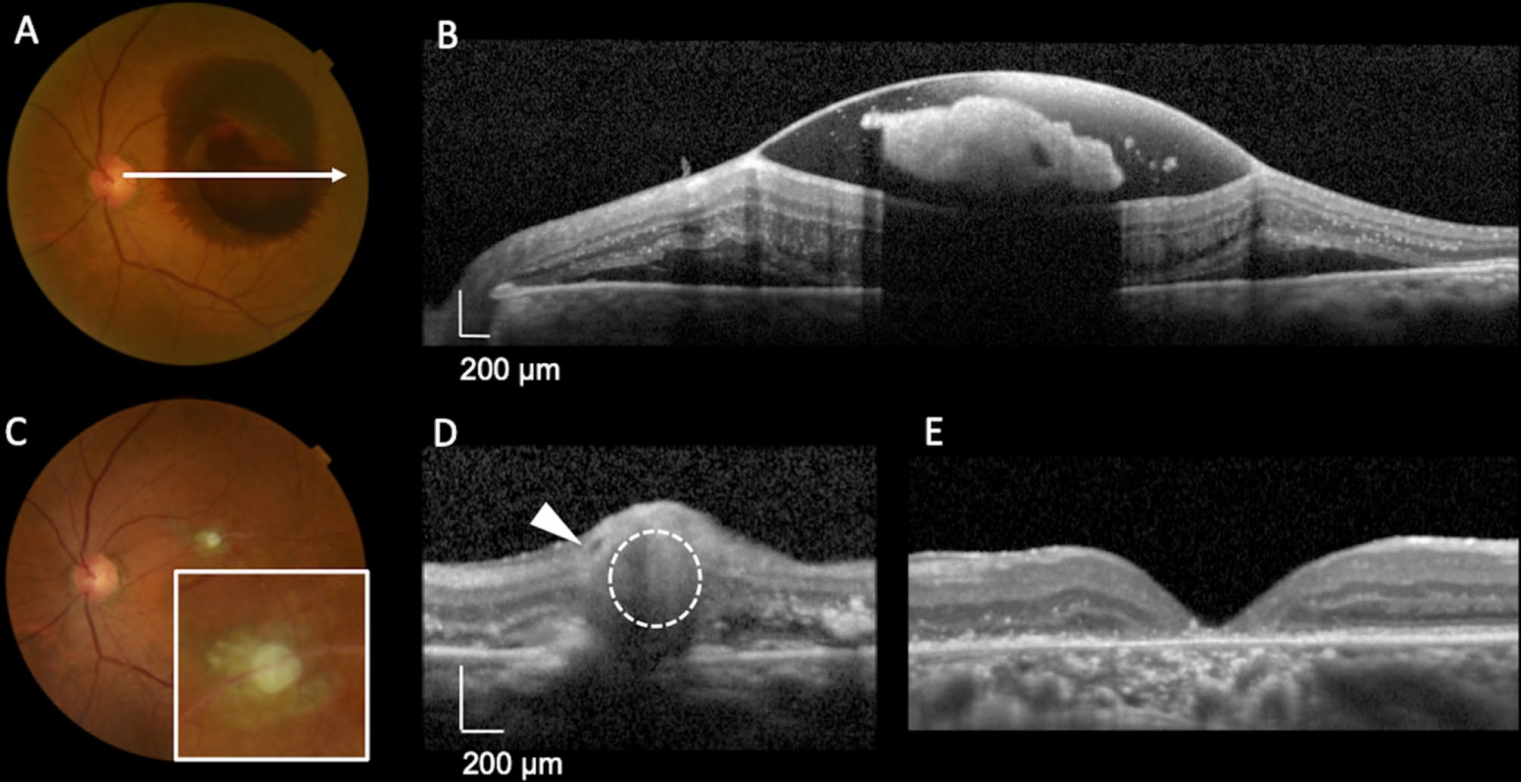
Representative case involving a 74-year-old woman with a ruptured retinal arterial macroaneurysm posterior to the affected artery (posterior type) in the left eye. At the initial visit, the visual acuity was 20/400. Optical coherence tomography (OCT) shows sub-inner limiting membrane (ILM) hemorrhage and a relatively large amount of subretinal hemorrhage (**B**). The thickness of the foveal subretinal hemorrhage was 287 μm. At 5 days after onset, we performed pars plana vitrectomy with ILM peeling, direct photocoagulation of RMA, and sulfur hexafluoride gas tamponade. OCT performed at the time of substantial resolution of the hemorrhage (**D**) shows RMA (dotted circle) located posterior to the artery (arrow), and color fundus photography (**C**) shows the organized white RMA located posterior to the affected retinal artery without obscuring its course. Thus, it was classified as the posterior type. At 24 months after onset, the length of the ellipsoid zone band defect at the fovea was 1725 μm (**E**), and the visual acuity was 20/800.

Figures 2, 3, 4 and 5 show the initial and final clinical findings according to the intraretinal RMA locations. At the initial visit, the logMAR VA for the posterior type (1.04 ± 0.58) was not significantly different from that for the lateral type (0.93 ± 0.66), although it was significantly poorer than that for the anterior type (0.49 ± 0.40; *P* = 0.005). The thickness of subretinal hemorrhage for the posterior type (291 ± 139 μm) was greater than that for the lateral (125 ± 111 μm; *P* = 0.016) and anterior (124 ± 153 μm; *P* = 0.006) types. There were no significant differences in the foveal thickness among the three types.

The final logMAR VA showed a significant positive association with the length of the EZ band defect at the final visit (*P* < 0.001). At the final visit, the length of the foveal EZ band defect was greater for the posterior type (780 ± 570 μm) than for the anterior type (236 ± 348 μm; *P* = 0.005). The final logMAR VA for the posterior type (0.59 ± 0.39) was poorer than that for the anterior type (0.14 ± 0.16; *P* < 0.001). The length of the foveal EZ band defect and the logMAR VA for the lateral type were not significantly different from those for the other two types.

## Discussion

In this study, we used OCT and CFP to examine the three-dimensional intraretinal locations of ruptured RMAs relative to the affected retinal artery and classified the lesions as anterior (anterior to the affected artery), lateral (at the same level as the affected artery), and posterior (posterior to the affected artery) RMAs (Supplementary Fig. 1). We then found that the positions of RMAs affected the initial VA, pattern of hemorrhage, degree of future damage to the foveal photoreceptors, and visual prognosis (Figs. 2, 3, 4, 5).

Histologically, retinal vessels are located in the inner retina and nourish the neuroglial cells in the corresponding retinal layers. In clinical practice involving patients with chorioretinal diseases, preretinal, intraretinal, and subretinal hemorrhages can be differentiated according to the visibility of the retinal vessels traveling in the inner retina. Previous OCT examinations of the retinal vessels showed that retinal arteries ran straight in the inner retina without changing their own depth even at arteriovenous crossings, pushing the veins anteriorly or posteriorly^14^. In this study, we accordingly investigated the three-dimensional relationship between ruptured RMAs and the affected retinal arteries in detail, using both OCT scans and CFP images.

Retinal macroaneurysm is clinically defined as an aneurysm arising within the first three branches of the central retinal artery^2^. However, the precise pathologies have not been well determined. Previous studies involving trypsin digestion showed various types of aneurysmal changes. Among the suggested types, the blowout aneurysm involved linear splits along the vessel wall, through which the aneurysmal sac protruded^15^. Pathological changes occurring in systemic arteries and aortic aneurysms and dissection, which are caused by the formation of cracks in the arterial wall from the intima to the tunica media, are well known^16,17^. The presence of retinal blowout aneurysms, pathologies of systemic arterial diseases, and lack of significant bias in the present study (anterior type, 44%; lateral type, 20%, and posterior type, 34%) might suggest that the accidental cracks in the retinal arterial walls would be involved in the initiation of the pathological changes associated with RMAs.

The present study showed that the morphological and functional prognoses of the fovea may differ according to the intraretinal locations of ruptured RMAs (Figs. 3, 4, 5, Table 3). We speculated that, because anterior RMAs occur between the arterial wall and ILM, the tension of ILM may tend to restrict the extent of rupture. The longitudinal elasticity of ILM (Young's modulus) is reported to be approximately 3000 to 30,000 times greater than that of the sensory retina^18^. In contrast, RMAs ruptured posterior to the arteries are less affected by ILM; thus, the extent of rupture may be larger and the subretinal hemorrhage may be more extensive. In cases with a larger amount of subretinal hemorrhage requiring a longer time to absorb, the foveal photoreceptors would be more susceptible to damage because of possible mechanisms such as reduced nutrition from the choroidal side^19^, oxidative stress^20^, iron toxicity^21^, and tearing of the outer segments of the photoreceptors by fibrin or fibrin degradation products^22^. These may have been involved in the more severe foveal photoreceptor damage and poorer visual prognosis for the eyes with posterior RMAs.

Recently, Doi et al. reported the results of vitrectomy using subretinal injection of t-PA in 23 eyes with submacular hemorrhage due to RMA rupture^11^. The authors used swept-source OCT to detect intraretinal hemorrhage and found that patients with preoperative intraretinal hemorrhage (74%) had a poorer postoperative VA than did those without hemorrhage (26%). However, the authors did not describe the mechanism underlying the variations in the degree of hemorrhage. Moreover, because RMAs rupture in the inner retina, the rate of intraretinal hemorrhage might be inherently higher. The present study using OCT, and another previous study, found much higher rates of intraretinal hemorrhage^23^. However, we cannot deny the possibility that the amount of intraretinal hemorrhage would be positively correlated with that of subretinal hemorrhage. In patients with RMA rupture, intraretinal hemorrhage could result from the retrograde flow of subretinal hemorrhage into the retina at the center of the fovea^11^.

Several treatment strategies have been reported for ruptured RMAs^5–10,24–26^. Among these, direct photocoagulation of RMAs is often performed because it is relatively easy and the therapeutic effect is not low^5,6^. On the basis of our OCT findings, we consider that direct photocoagulation is not suitable for all patients with RMA because the posterior type is located below the affected artery and consequently difficult to coagulate. On the other hand, the anterior and lateral types can be coagulated more efficiently, which results in suppression of exudative changes. Thus, it would be necessary to pay attention to the relative position of RMA and the retinal artery at the time of planning treatment, especially direct photocoagulation. The possibility of increased risk of retinal arterial occlusion when the affected retinal artery is also coagulated is another concern in the treatment of posterior RMAs.

As limitations of this study, we noted several factors. First, because of the retrospective design, the treatment regimen was not uniform. However, we consider that the final VAs were not significantly biased according to the treatment protocols, and there were no significant associations between the treatment methods and intraretinal locations of RMAs (data not shown). Second, because we only measured the subretinal hemorrhage “height” using two B-scans sectioning the fovea horizontally and vertically, we could not accurately assess the total amount of subretinal hemorrhage, given that these hemorrhages probably spread two-dimensionally in the retinal plane and tangentially to the retinal plane. Third, accurate evaluation of the intraretinal location of RMA soon after onset is difficult because of the significant amount of associated hemorrhage. Therefore, in this study, we assessed the intraretinal location when the hemorrhages were substantially absorbed. Although we believe that the presumed intraretinal location is useful for selecting an appropriate treatment protocol even in the acute phase, the rupture depth evaluated at this time may not be very useful for predicting the visual prognosis in the acute phase. Lastly, the effects of age-appropriate cataracts and the results of cataract surgery on final VA cannot be completely ignored.

Despite the limitations, the results of the present study suggest that RMA may occur at various depths in the affected artery, and that variations in the intraretinal depth may affect the hemorrhagic pattern, volume of subretinal hemorrhage, and visual prognosis. These findings may be useful for selecting effective treatments. However, a prospective study with a uniform treatment regimen is necessary to confirm the reproducibility of the results of this study.

## Supplementary Information

Supplementary Information.

## Data Availability

The datasets generated during and/or analyzed during the current study are available from the corresponding author on reasonable request.
