# Supplementary material for: Three-dimensional locations of ruptured retinal arterial macroaneurysms and their associations with the visual prognosis
Source: Sci Rep. 2022 Jan 11;12:503. doi: 10.1038/s41598-021-04500-4 (PMC8752622; doi:10.1038/s41598-021-04500-4)
Supplement: Supplementary file 1 — Supplementary Information. [file 41598_2021_4500_MOESM1_ESM.pdf]

Three-Dimensional Locations of Ruptured Retinal Arterial Macroaneurysms and their Associations with the Visual Prognosis

SAORI SAKAGUCHI, YUKI MURAOKA*, SHIN KADOMOTO, SOTARO OOTO, TOMOAKI MURAKAMI, NAOMI NISHIGORI, MASAHARU ISHIKURA, MASAHIRO MIYAKE, MANABU MIYATA, AKIHITO UJI, AKITAKA TSUJIKAWA

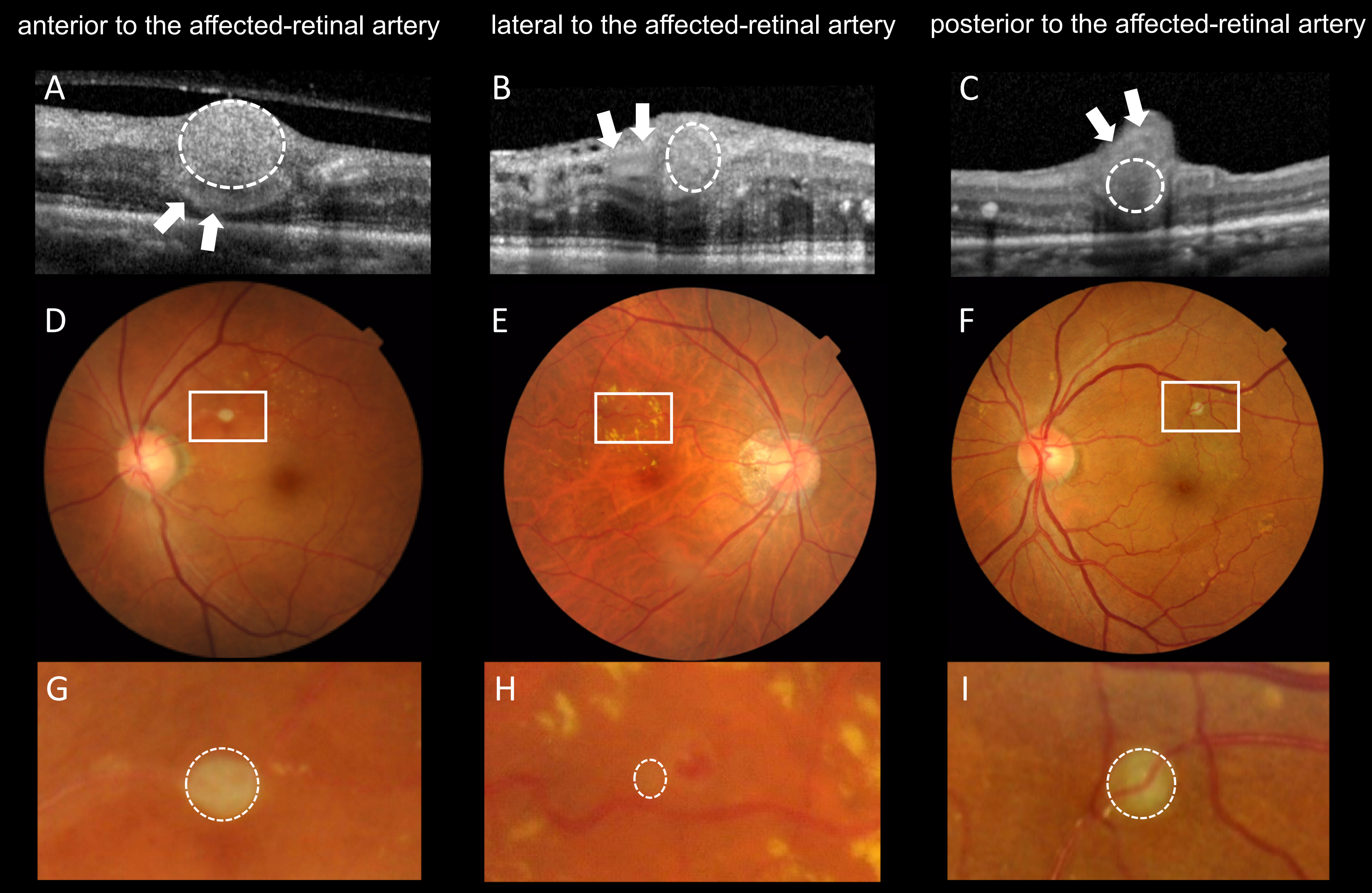
anterior to the affected-retinal artery
lateral to the affected-retinal artery
posterior to the affected-retinal artery
A
B
C
D
E
F
G
H
I

Supplementary Figure 1. Evaluation of the position of a ruptured retinal arterial macroaneurysm relative to the affected retinal artery using optical coherence tomography and color fundus photography

A, B, C. Optical coherence tomography (OCT) images of the ruptured retinal arterial macroaneurysm (RMA, dotted circles) and the affected retinal artery (white arrows)

D, E, F, G, H, I. Color fundus photography (CFP) images of RMA (dotted circles), with magnifications of the square

A, D, G. RMA anterior to the affected retinal artery (anterior type)

B, E, H. RMA at the same level as the affected retinal artery (lateral type)

C, F, I. RMA posterior to the affected retinal artery (posterior type)

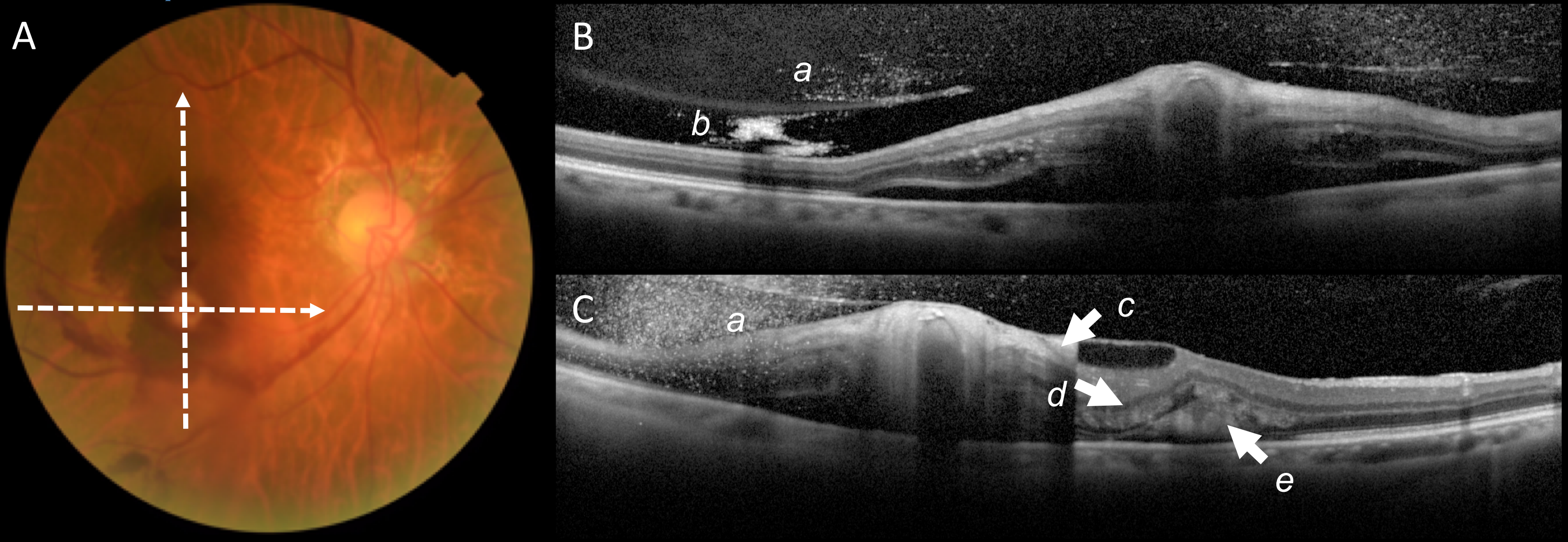

a. vitreous hemorrhage b. preretinal hemorrhage c. sub ILM hemorrhage d. intraretinal hemorrhage e.subretinal heorrhage

Supplementary Figure 2. Different hemorrhage patterns in an eye with a ruptured retinal arterial macroaneurysm using optical coherence tomography; a: Vitreous hemorrhage (anterior to the posterior vitreous membrane), b: Preretinal hemorrhage (anterior to the inner limiting membrane and posterior to the posterior vitreous membrane), c: Sub-inner limiting membrane hemorrhage (confined below the inner limiting membrane), d: Intraretinal hemorrhage (inside the sensory retina), e: Subretinal hemorrhage (beneath the sensory retina).
